# Macrophage function in the elderly and impact on injury repair and cancer

**DOI:** 10.1186/s12979-021-00215-2

**Published:** 2021-01-13

**Authors:** L Duong, HG Radley, B Lee, DE Dye, FJ Pixley, MD Grounds, DJ Nelson, C Jackaman

**Affiliations:** 1 Curtin Medical School, Curtin Health Innovation Research Institute, Faculty of Health Sciences, Curtin University, Kent Street, 6102 Bentley, Western Australia Australia; 2 School of Biomedical Sciences, University of Western Australia, 6009 Nedlands, Western Australia Australia; 3 School of Human Sciences, University of Western Australia, 6009 Nedlands, Western Australia Australia

**Keywords:** Macrophages, Age‐related diseases, Inflammation, Cancer, Injury repair

## Abstract

Older age is associated with deteriorating health, including escalating risk of diseases such as cancer, and a diminished ability to repair following injury. This rise in age-related diseases/co-morbidities is associated with changes to immune function, including in myeloid cells, and is related to immunosenescence. Immunosenescence reflects age-related changes associated with immune dysfunction and is accompanied by low-grade chronic inflammation or inflammageing. This is characterised by increased levels of circulating pro-inflammatory cytokines such as tumor necrosis factor (TNF), interleukin (IL)-1β and IL-6. However, in healthy ageing, there is a concomitant age-related escalation in anti-inflammatory cytokines such as transforming growth factor-β1 (TGF-β1) and IL-10, which may overcompensate to regulate the pro-inflammatory state. Key inflammatory cells, macrophages, play a role in cancer development and injury repair in young hosts, and we propose that their role in ageing in these scenarios may be more profound. Imbalanced pro- and anti-inflammatory factors during ageing may also have a significant influence on macrophage function and further impact the severity of age-related diseases in which macrophages are known to play a key role. In this brief review we summarise studies describing changes to inflammatory function of macrophages (from various tissues and across sexes) during healthy ageing. We also describe age-related diseases/co-morbidities where macrophages are known to play a key role, focussed on injury repair processes and cancer, plus comment briefly on strategies to correct for these age-related changes.

## Background: macrophage function and healthy ageing

Macrophages are numerically abundant phagocytic cells found in most tissues throughout the body [1, 2]. They play a key role in maintaining homeostasis as they can remove deleterious senescent cells that increase during ageing [3]. They are also highly plastic and can acquire many functional states in response to local environmental signals ranging from pro-inflammatory, anti-tumorigenic to anti-inflammatory, pro-tumorigenic or wound healing macrophages [4–6]. Historically, these cells have been generally classified into M1 and M2 respectively, however more recent studies have suggested that this broad classification is too simplified to apply to dynamic *in vivo* studies [7–9]. Nomenclature based on macrophage function is a more logical, physiologically relevant approach, and is further outlined elsewhere [10]. Therefore, here we discuss the inflammatory function of these cells in relation to *in vivo* temporal dynamics and their functional response.

Macrophages can be activated early in a response to pro-inflammatory stimuli, such as bacterial lipopolysaccharide (LPS) during bacterial infection [11]. Macrophages can also be activated early in a response following sterile injury and necrosis due to the release of ‘danger signals’ such as high-mobility group box-1 [12]. Following early activation, macrophages secrete pro-inflammatory cytokines and chemokines that induce inflammation and promote immune cell recruitment. Secreted cytokines include IL-6, IL-12, and TNF [5, 11, 12] whilst secreted chemokines include C-X-C motif ligand (CXCL9) and CXCL10, which recruit helper CD4^+^ T cells and effector CD8^+^ T cells [13]. Macrophages can also be activated later in a response leading to an anti-inflammatory or reparative phenotype [14]. These reparative macrophages secrete cytokines such as IL-10 and TGF-β1 that are critical for wound healing [14] and recruit regulatory CD4^+^ T cells (Tregs) via release of C-C motif ligand 17 (CCL17), CCL22, CCL24 and CXCL13 [5, 14]. While these factors stimulate wound healing and injury repair in mice and humans [13, 14], they can also influence angiogenesis and tumor growth [5].

Studies suggest that early, pro-inflammatory macrophages can transition into anti-inflammatory, reparative macrophages [15, 16]. This functional transition may occur following phagocytosis [15] or in response to inflammatory microenvironmental changes following the recruitment of other immune cells such as Tregs [16, 17], as depletion of Tregs impairs this process [18, 19]. Tregs secrete regulatory cytokines, such as IL-4, IL-10 and TGF-β1, which can modulate macrophage transition from pro-inflammatory to reparative function. The temporal dynamics of the immune cell landscape is tightly regulated to allow normal resolution of inflammation; specifically, as reparative macrophages become increasingly abundant their secreted factors inhibit the recruitment and function of pro-inflammatory cells [20–22]. Furthermore, pro-inflammatory TNF is inhibited by regulatory IL-10 due to cross-regulation of the Janus kinase/signal transducers and activators of transcription (JAK/STAT) pathway [23]. Macrophage transitioning from pro-inflammatory to reparative phenotype may be dysregulated with ageing. For example, studies show that macrophages from aged hosts having diminished ability to phagocytose apoptotic cells, including neutrophils; this is associated with impaired inflammatory resolution [24, 25].

### Macrophage origin, tissue site and ageing

Ontogeny studies in young hosts have shown that macrophages can be derived either from immature bone marrow myeloid progenitors that are released into the blood as monocytes and traffic to tissue sites where they differentiate into macrophages [26, 27]; or as tissue-resident macrophages that develop early during embryogenesis as a separate lineage to bone marrow-recruited cells and self-proliferate locally [27–29]. The relative proportions of tissue resident to bone marrow-derived macrophages varies between tissues and may impact their function [30]. The function of tissue-resident macrophages during homeostasis can be dependent on the tissue site [31]. For example, brain-resident microglia support neuron survival, alveolar macrophages play a role in immune surveillance and adipose macrophages help control insulin sensitivity and adaptive thermogenesis [31]. During an acute or chronic inflammatory response, further monocytes can be recruited from the bone marrow or spleen and contribute to the tissue-resident population [26].

Whether macrophage origin influences their response during ageing or whether context-dependent environmental changes at the tissue site is the driver for age-related changes is currently unknown. However, studies suggest that age-related functional changes may be tissue site-specific [12]; these changes could be due to macrophage origin. For example, we have previously examined macrophages in healthy young versus aged C57BL/6J female mice (24–28 months) and found there was an increase in IL-10^+^ macrophages in the spleen and bone marrow of healthy aged mice [32]. In young hosts, macrophages at these sites are predominantly of monocyte origin [29] and can supply macrophages to the tumor microenvironment or during injury repair [33, 34]. Others have shown elevated anti-inflammatory macrophages in the eye [35], lung [36] and muscle [37] of healthy Balb/c and C57BL/6J mice during ageing. Conversely, pro-inflammatory macrophages increase in the liver, brain and adipose tissue during healthy ageing in mice [38–40]. During ageing there are also changes to the tissue microarchitecture, for example loss of marginal zone macrophages in the spleen [41] leading to altered local interactions between macrophages, neutrophils and T cells [42], which could further impact immune regulation. Similarly, brain-resident microglia from aged (27–28 months) male and female C57BL/6J mice increase in soma volume but reduce the length of their cell processes, limiting their capacity to interact with and support neuron survival during homeostasis [43]. It is possible that changes to cell size also occurs in bone marrow-derived monocytes/macrophages and haematopoietic stem cells similar to that observed in bone marrow mesenchymal stromal cells [44].

These varying responses could also be due to tissue-specific differences in macrophage-related responses between males and females during ageing. Studies have suggested that ‘sex is a biological variable that should be considered in immunological studies’ [45]. Females tend to have a more robust immune system, heightened immune response and better resistance to infection than males [46], which is likely to be further impacted during ageing. Furthermore, it is now recognised that sex chromosomes (via extent of inaction of the second X chromosome) can also directly exert effects on immune function, in addition to the role of sex hormones [47]. Inflammageing, characterised by elevated IL-6, is higher in male compared with female humans [48]. Elderly men also display increased levels of circulating inflammatory CD14^+^ monocytes compared to females [49]. However, tissue macrophages were increased in hearts of elderly (50–68 years) female compared to male humans, which was associated with a pro-inflammatory shift during ageing [50]. Similarly, aged female C57BL/6J mice also exhibit greater microglial-associated neuroinflammation in comparison to male mice [51]. Interestingly, macrophage turnover in the peritoneal cavity is also influenced by age and sex, which subsequently impacts response to pneumococcal peritonitis [52]. Therefore, it is possible that changes to macrophage function during ageing could be related to differences in macrophage origin and turnover between females and males.

*In vitro* studies suggest that macrophages from elderly humans and mice exhibit an altered response to stimuli, which may also depend on the tissue site, sex and location these cells have been isolated from. For example, we have previously published that in response to tumor-derived factors elderly-derived peritoneal macrophages from female C57BL/6J mice (aged 24–28 months) and Balb/c mice (18 months) secrete increased levels of TGF-β1 and IL-4 compared to young mice [32, 53]. Similarly, Smallwood et al. [54] suggested that aged male Balb/c peritoneal macrophages (14–15 months) were in a pre-activated basal state that enhanced their response to LPS. Moreover, bone marrow-derived macrophages from aged female C57BL/6J mice (16–22 months) and elderly humans cultured with LPS exhibited increased TNF and IL-6 production [55, 56]. However, splenic macrophages from aged female Balb/c mice (18–20 months) stimulated with LPS released lower levels of IL-1β and TNF [57]. Furthermore, we and others have shown that splenic macrophages from aged female C57BL/6J mice (20–24 months) exhibit reduced phagocytosis and proliferative capacity [42, 58]. These altered responses may be due to impaired STAT-1, p38 and JNK mitogen-activated protein kinases (MAPK) signalling in elderly-derived macrophages [59].

These results may also be explained by recent studies in young mice and humans demonstrating that macrophages can undergo ‘training’ resulting in a heightened pro-inflammatory phenotype or immune tolerance following re-exposure to the same or another stimulus such as LPS [60, 61]. Training can occur systemically in circulating monocytes and monocyte-derived macrophages [62, 63]. Tissue-resident macrophages can also undergo training, with brain-resident microglial cells shown to be trained after systemic exposure to a single dose of LPS or tolerized after repeated exposure to LPS [64]. Whilst tissue-resident alveolar macrophages trained following respiratory viral infection subsequently contributed to improved anti-bacterial immunity through rapid induction of CXCL1 and CXCL2 [65].

Interestingly, the enhanced or altered macrophage cytokine production that occurs during ageing in response to stimuli is similar to that observed in the training studies, which were carried out in young hosts [66, 67]. Indeed, during ageing there is an increase in stimuli associated with training. For example, intestinal permeability increases in aged C57BL/6J mice (18–22 months) leading to microbial products entering the bloodstream [56]. Similarly, LPS binding protein (a surrogate marker for bacterial products) is elevated in the serum of elderly compared to young humans. Additionally, damage associated molecular pattern molecules (DAMPs) accumulate in mice and humans as they age [68]. Further, recent studies have described that trained immunity in young mice leads to increased myeloid lineage cells and can occur in myeloid precursors in the bone marrow [69]. During ageing a similar shift towards a myeloid cell lineage occurs, and it is possible that the ageing microenvironment leads to training [66, 67]. Similarly, alterations in intestinal permeability, combined with inflammageing, may impact blood-brain barrier permeability and the function of brain-resident microglial populations. However, additional studies are required to confirm this and whether age-related macrophage training impacts responses to injury repair and cancer.

### Macrophage metabolic function and ageing

Macrophage inflammatory responses involve metabolic reprogramming, switching from oxidative phosphorylation mediated by mitochondrial function in resting cells, towards glycolysis in activated cells [70]. The change in energy metabolism enables pro-inflammatory macrophages to perform effector functions, such as increased production of inflammatory mediators e.g. IL-1β, TNF and IFN-γ [70, 71]. In contrast, anti-inflammatory macrophages are supported by both oxidative phosphorylation and glycolysis [72, 73]. It is possible that changes to macrophage metabolism during ageing impacts their activation and function [74, 75].

It has been proposed that mitochondrial dysfunction occurs during ageing due to reduced synthesis of nicotinamide adenine dinucleotide (NAD) [76, 77], which is a cofactor of key enzymes in the TCA cycle, glycolysis and oxidative phosphorylation [78]. Declining levels of NAD+ and dysregulation of regulatory pathways, such as the kynurenine pathway could impact macrophage function during ageing as these are key metabolites involved in macrophage activation [79]. Furthermore, CD38 an enzyme involved in degradation of NAD increases with age [76]. Genetic ablation or pharmaceutical inhibition of CD38 can reverse mitochondrial dysfunction and reduce inflammatory cytokines in human monocyte/macrophages and in mice [80, 81]. Interestingly, CD38 is highly expressed in pro-inflammatory macrophages [80]. Therefore, it is possible that increased circulating levels of inflammatory factors in the ageing microenvironment induce CD38 expression, contributing to metabolic dysregulation and in turn promoting the inflammatory function of macrophages in the elderly. However, in contrast Fei et al. 2016 [82], showed that metabolic reprogramming of oxidative phosphorylation to glycolysis was impaired in bone-marrow derived macrophages from C57BL/6J mice (18–22 month, sex not stated). This could also be related to tissue-specific metabolic changes during ageing (reviewed by [74, 75], further studies are required to determine whether similar changes occur during injury repair and cancer.

### Macrophages and myeloid‐derived suppressor cells

It is also possible that the changes to macrophages that occur during ageing are due to early release of related precursor cells from the bone marrow known as myeloid-derived suppressor cells (MDSCs) [83, 84]. MDSCs are generally classified into two subsets, monocytic MDSCs or polymorphonuclear MDSCs [85]. Phenotypically, monocytic-MDSCs have a greater resemblance to monocyte/macrophages and polymorphonuclear-MDSCs have greater resemblance to polymorphonuclear cells. Further classification is beyond the scope of this review and discussed elsewhere [85]. Generation and expansion of pro-tumor MDSCs is mediated by colony stimulating factors (CSFs e.g. granulocyte-CSF, macrophage-CSF and granulocyte/macrophage-CSF) and pro-inflammatory factors (e.g. IFN-γ and IL-6). Subsequently, TGF-β1 and IL-13 produced by MDSCs can further augment their immunosuppressive capabilities [84].

MDSCs can mediate an increase in Tregs via the release of arginase, IL-6 and IL-10, consequently leading to elevated secretion of immunosuppressive factors by Tregs (e.g. IL-10, TGF-β1, IL-4) [84]. MDSCs can also suppress T cell activation and proliferation [86, 87], however, in a murine breast cancer model MDSCs were shown to be less immunosuppressive compared to tumor-associated macrophages [87]. MDSCs expressed lower levels of anti-inflammatory *Arg1, Il-10, Ccl17*, and *Ccl22*, but produced higher levels of angiogenic factors compared to tumor-associated macrophages [87]. Studies have also shown that MDSCs can differentiate into tumor-associated macrophages [86, 87]. Furthermore, hypoxic regions found in tumors can promote MDSC differentiation into tumor-associated macrophages [86], and a higher proportion of anti-inflammatory tumor-associated macrophages are associated with these hypoxic regions [86, 88]. We and others have shown that MDSCs increase in spleen, bone marrow and lymph nodes of healthy aged C57BL/6J female mice [32], albeit based on expression of CD11b^+^GR-1^+^ cells) and in peripheral blood in humans [89]. This may be due to inflammageing in combination with a shift towards a myeloid cell lineage that occurs in the bone marrow during ageing [83, 90]. As described, this is likely to impact macrophage responses during ageing, and following injury repair and cancer.

### Macrophages in musculoskeletal injury repair and ageing

**Fig. 1:**
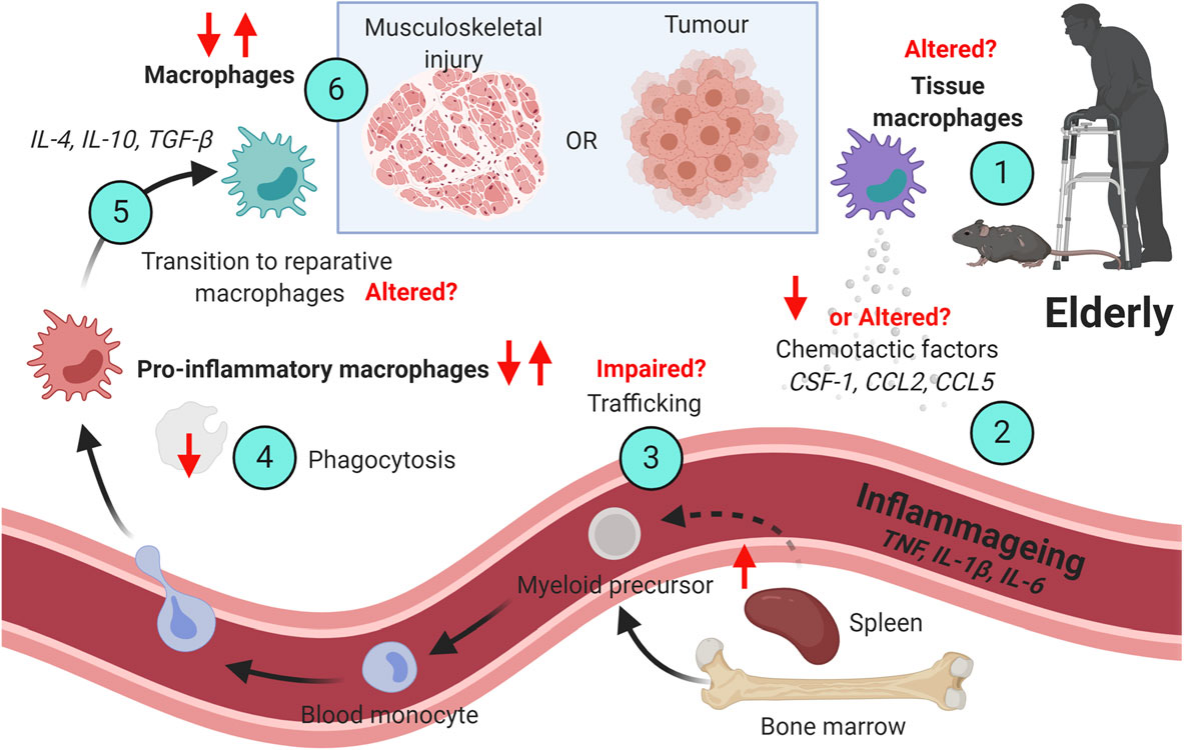
Age-related changes to macrophages during musculoskeletal repair and cancer . Following musculoskeletal injury or tumor growth, there may be changes associated with tissue-resident macrophages and tissue site during ageing (1). This may lead to decreased or altered chemotactic signals (2), driven by factors such as CSF-1, CCL2 or CCL5. Bone marrow and splenic myeloid cells are increased during ageing (3) and can supply macrophages to the tumor and injury site, however this could be further impacted by inflammageing factors such as TNF, IL-1β and IL-6. Macrophages during ageing generally display reduced capacity for phagocytosis (4). This may lead to altered transitioning from pro-inflammatory to reparative macrophages (5), which can also be driven by factors such as IL-4, IL-10 and TGF-β1. Studies are conflicting during ageing as to whether there are increased or decreased macrophages following musculoskeletal injury or during tumor growth (6). Age-associated changes are shown as increased (↑); decrease (↓); unknown (?). This Figure was created with BioRender.com

For effective repair following musculoskeletal injury in young hosts, pro-inflammatory macrophages are found in the area of damage early post injury in both humans and mice [91, 92]. For example, in young mice, neutrophils appear within minutes at the site of injury and chemoattract further monocytes/macrophages that are then a major source of chemoattractants for myoblasts [93]. Both neutrophils and monocytes gather at the damaged tissue by 8 h post muscle injury, and from 24-48 h macrophages become the most abundant cell type present [92, 94]. Accompanying neutrophils assist macrophages in the phagocytosis of cell debris and induce local inflammation [92]. Upon entry to the necrotic area, monocytes begin to phagocytose injured tissue [15]; this inflammatory environment can cause monocytes to differentiate into pro-inflammatory macrophages [15]. Studies have shown that early pro-inflammatory macrophages can influence myogenic cell proliferation via secretion of pro-inflammatory cytokines such as IL-6, however this inhibits myoblast differentiation [95]. Following phagocytosis of debris, the induction of reparative macrophages promotes muscle repair [15]. These reparative macrophages found later during the repair process enhance the differentiation and fusion of myoblasts to form myotubes that later fuse to reseal damaged myofibres, mainly through secretion of anti-inflammatory factors, such as TGF-β1 [15, 92]. A similar process occurs during bone fracture healing, with similar temporal macrophage cross-talk occurring with osteoblasts/osteoclasts rather than myocytes (reviewed by [96]. It is well known that musculoskeletal repair is delayed in elderly humans and mice and this is strongly associated with an altered inflammatory responses as shown by a cross-transplantation study of muscle grafts between young (3 months) and very old (27–29 months) C57BL/6J female mice [97]. The extent to which these inflammatory changes in damaged elderly tissues are due to altered levels of chemotactic factors produced by the damaged tissues, or reflects impaired and/or reflects dysregulated macrophage function is discussed below and summarised in Fig. 1.

Comparison of skeletal muscle macrophage levels in young and elderly healthy humans showed that elderly subjects have higher numbers of reparative macrophages (based on increased *CD206* expression) than younger subjects [98]. It is possible that this increase in reparative macrophages is a compensatory response to increasing levels of skeletal muscle pro-inflammatory molecules (e.g. TNF) observed in muscles of elderly patients (81 years old) male compared to young patients (23 years old) [99], and in muscles from aged C57BL/6J male mice [100]. Interestingly, following cardiotoxin-induced injury, aged male C57BL/6J mice (24 months) showed decreased infiltration of macrophages into damaged muscle [101], which was associated with decreased chemokine secretion (particularly interferon-gamma inducible protein-10). This is similar to the delayed inflammatory infiltration previously observed with cross-transplantation young-old studies in aged female C57BL/6J mice [97], suggesting that the ageing microenviroment impacts chemotaxis [102]. It is possible that macrophage dysregulation during ageing also impacts cross-talk with other key immune cells required for repair. For example, decreased Treg infiltration in muscle following cardiotoxin injury in aged C57BL/6J male mice (> 20 months) was also associated with delayed repair [103]. Furthermore, reduced vascularity and increasing levels of fibrosis in the skeletal muscle are also thought to contribute to a dysregulated inflammatory response and reduced infiltration by macrophages in aged mice [102]. In contrast, in a bone-fracture healing model, macrophages from aged C57BL/6J mice (24 months old, sex not stated) exhibited a heightened inflammatory signature and preventing macrophage infiltration improved healing outcomes [104]. This is similar to the heightened inflammatory signature observed in elderly hip fracture patients and thought to be associated with dysregulated inflammatory monocyte/macrophages [66, 105].

### Macrophages in cancer and ageing

Macrophages can make up a large percentage of the tumor burden and are generally associated with poor prognosis [106, 107]. It has been proposed that the macrophage activation states required for injury repair are similar to those seen in cancer and cancer has been described as “the wound that does not heal” [108]. Whilst few studies have investigated macrophage function and their influence on other immune cells in cancer in the elderly, we may be able to draw parallels from the injury repair studies described above (summarised in Fig. 1). For example, tumor evolution starts with chronic inflammation wherein pro-inflammatory macrophages producing free radical species and nitric oxide promote neoplastic transformation [5, 6]. Monocytes and monocyte-derived macrophages can be recruited to tumors in response to chemotactic signals, such as CSF-1 [109, 110], CCL2 [110–112] and CCL5 [112] released by tumor cells and stroma [113]. Once established, tumor-derived factors drive macrophages towards an immunosuppressive phenotype mediated through IL-4, IL-10 and TGF-β release [5, 6] similar to reparative macrophages during injury repair [92]. Similarly, during initial stages of lung carcinogenesis in mice, tumor-associated macrophages were mainly pro-inflammatory (expressing IL-12 and IL-1β), and later transitioned to promote angiogenesis and tumor growth [114]. We have also previously observed a similar (albeit “incomplete”) shift in macrophage phenotype (based on expression of TNF and IL-10) during tumor growth in young female C57BL/6J mice with mesothelioma tumors [115].

Previous studies suggest that macrophages provide further support for tumor growth in the aged microenvironment. For example, peritoneal macrophages from aged (24 months) male and female C57BL/6N mice have reduced direct anti-tumor cytotoxic activity [116]. Furthermore, we have shown that peritoneal macrophages that originate from either aged (26 months) C57BL/6J female mice or Balb/c female mice (18 months) stimulated with tumor-derived factors increased production of TGF-β1 and IL-4, relative to macrophages from young mice [32, 53]. In a prostate cancer model, transcriptomic analysis of the tumor microenvironment revealed an increase in the expression of genes associated with pro-tumorigenic macrophages, such as *Arg1 Cd163, Mrc1, Retnla, Lyve1*, in aged (20–24 months) compared to young male C57BL/6J mice [117]. Similarly, in human prostate cancer there was an increase in expression of *CD163*, a monocyte and macrophage-specific scavenger receptor, in elderly patients which corresponded with poorer survival [117].

Several studies have also shown that T cells and macrophages have altered function associated with ageing which correlates with tumor progression [58, 118, 119]. Tumor-associated macrophages displaying altered inflammatory function in the aged tumor microenvironment may influence subsequent recruitment of T cells [120]. For example, Tregs have been shown to increase in lymphoid tissues of aged mice and humans, yet display decreased infiltration in tumors from elderly cancer patients [121, 122]. Provinciali and his colleagues also reported decreased infiltration of CD4^+^ and CD8^+^ T cells in mammary adenocarcinoma from aged (21–24 months) male Balb/c mice compared to young mice [119]. We have reported that CD8^+^ T cells in aged (22–24 months) tumor-bearing C57BL/6J female mice support tumor growth, suggesting that a shift towards a regulatory rather than cytotoxic phenotype occurs during ageing [123]. This could be due to cross-talk with macrophages as we have also recently shown that macrophages impair cytotoxic T lymphocyte function *in vivo* in the draining lymph nodes and tumor site of aged (20–24 months) tumor-bearing C57BL/6J female mice during immunotherapy [58]. Finally, in a mouse mammary adenocarcinoma model, MDSCs were associated with increased tumor susceptibility in aged (12 months) female BXD12 mice [124]. Depletion of MDSCs slowed tumor growth and partially restored T cell activity [124].

### Potential for targeting macrophages during ageing and age-related diseases

**Fig. 2:**
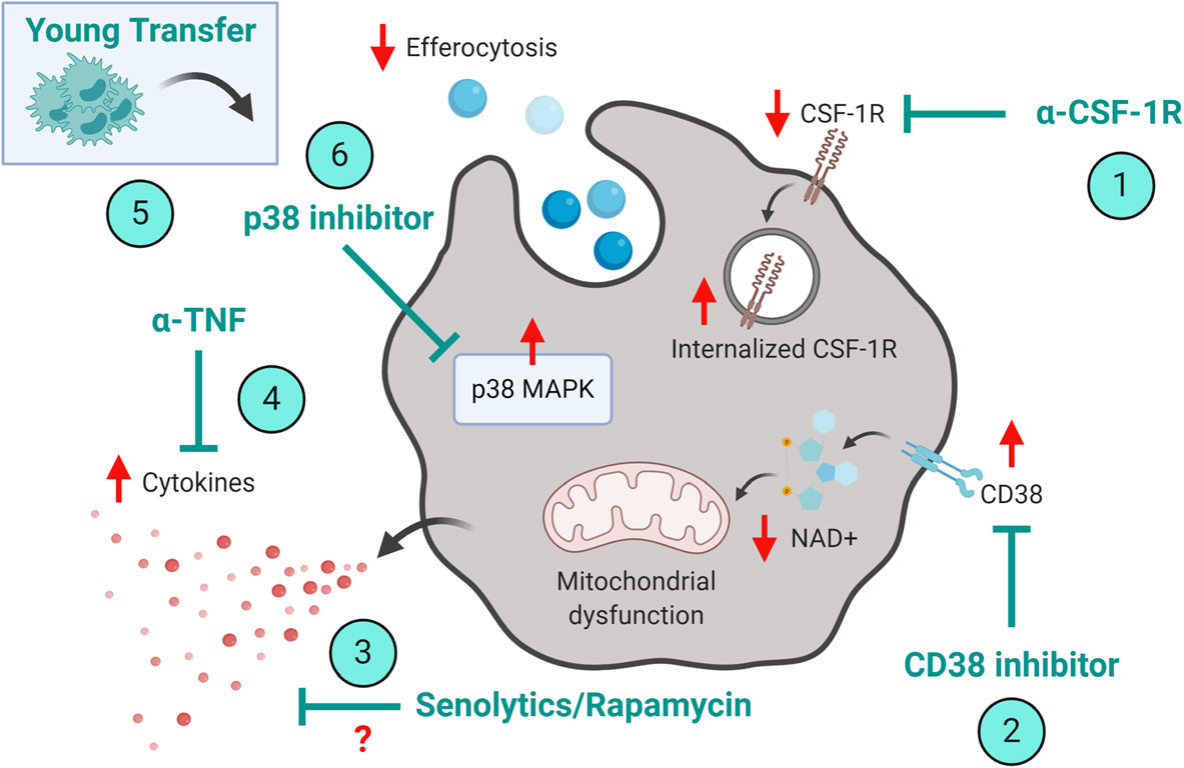
Strategies to target macrophages during age-related diseases. Potential strategies to target macrophages during ageing could include inhibiting recruitment via CSF-1R blockade (1), restoring metabolic function via inhibition of CD38 (2), targeting inflammageing via rapamycin/senolytics (3), anti-TNF (4), transfer of young serum/plasma/blood products (5), or reducing intracellular MAPK signalling leading to increased efferocytosis (6). Age-associated changes are shown as increased (↑); decrease (↓); unknown (?). This Figure was created with BioRender.com

Targeting of inflammation as a therapeutic approach accords with the interdisciplinary field of geroscience that aims to maintain and extend the duration of the healthy lifespan, as a strategy to reduce the impact of age-related conditions [125]. However, few studies have examined direct targeting of macrophages in the elderly as a therapeutic intervention to improve the outcomes of various chronic age-related diseases (summarised in Fig. 2), such as cancer and delayed tissue repair. A critical consideration is whether the chosen drug(s) target elderly cells with the same efficacy as seen in young hosts (considering that many pre-clinical studies are carried out using young adult animal models). For example, the elderly are often excluded from clinical trials due to reduced kidney and liver function which could impact drug pharmacokinetics and efficacy [126] yet the elderly might be a likely future target population for this drug intervention.

Recent studies in humans and mice that focussed on targeting tumor-associated macrophage infiltration via blockade of colony stimulating factor-1 receptor (CSF-1R) have shown promise [6, 127]. Yet downstream MAPK signalling is altered in elderly-derived macrophages, which could impact efficacy of CSF-1R inhibition in the elderly [59]. Furthermore, in healthy and tumor-bearing aged female C57BL/6J mice (20–23 months) we have observed downregulation and internalisation of CSF-1R on monocytes/macrophages (unpublished observations). It is possible that altered or reduced CSF-1R targeting in the elderly results in partial macrophage depletion which is beneficial in the aged setting. For example, in proof-of-principle studies we have shown that targeted, partial depletion of macrophages by anti-F4/80 antibody (approximately 40% reduction of F4/80^hi^ cells only) in aged tumor-bearing female C57BL/6J mice (20–24 months) improved response to IL-2/anti-CD40 immunotherapy, i.e. increased cytotoxic lymphocyte activity, reduced treatment-associated cachexia and tumor regression [58]. However, the same treatment worsened anti-tumor responses in young mice when macrophages were no longer present, highlighting the different roles of macrophages in anti-tumor responses in the ageing host.

Studies have suggested that compensatory regulatory mechanisms may exist in the elderly microenvironment [128], potentially due to inflammageing [90]. For example, following musculoskeletal injury in CCR2-deficient mice, decreasing pro-inflammatory monocyte/macrophages infiltration led to an increased pro-inflammatory microenvironment [129]. This could be counteracted by administration of reparative cytokines, as improved musculoskeletal repair was observed by administration of growth differentiation factor 3 in aged male C57BL/6J mice following cardiotoxin-induced injury (23 months old, [130]. Targeting recruitment of myeloid cells via CXCR2 can reduce infiltration of immature MDSCs [131]. Similarly, studies in young tumor-bearing mice have targeted MDSCs via all-trans retinoic acid leading to maturation of myeloid cells and downregulation of secreted factors such as reactive oxygen species via an ERK1/2 dependent mechanism [132]. However, this signalling pathway may also be impaired in MDSCs from aged mice, along with PI3K signalling [133]. Interestingly, depletion of MDSCs in aged (17–19 months, sex not stated) but not young C57BL/6J mice slowed tumor growth [128]. Furthermore, depletion of Treg cells in tumor-bearing mice led to a subsequent increase in MDSCs in aged but not young C57BL/6J mice [128], highlighting complex immune-mediated compensatory mechanisms that may exist in the elderly microenvironment.

Targeting of inflammageing via TNF blockade was effective in restoring responses to cancer immunotherapy [55] and as a strategy to reduce infection in aged mice [56]. Both of these studies showed that age-associated TNF was associated with dysregulated macrophage function in the aged mice. Similarly, young to old heterochronic parabiosis experiments or transfer of serum/plasma/blood products have been an effective strategy to dampen inflammageing and improve function in aged mice [134–136]. Also, as described above genetic ablation or pharmaceutical inhibition of CD38 can reverse mitochondrial dysfunction and improve inflammageing in aged (22–26 months) C57BL/6J male mice [74, 81]. Other strategies to broadly reduce inflammageing also include targeting of senescent cells and the mammalian target of rapamycin pathway, (reviewed in [137]. However, few studies have examined the direct impact of using senolytics or rapamycin to target macrophage function in age-related diseases. It is possible that reduction of inflammageing, rather than complete inhibition [137, 138], could be an effective strategy to modulate macrophage function in the elderly. This was highlighted in a recent study where elevated MAPK in elderly monocytes led to reduced efferocytosis and an increased pro-inflammatory response in a dermal model of acute inflammation in elderly humans [139]. Targeted reduction of MAPK in elderly monocytes, by using an oral p38 inhibitor, led to an increase in pro-resolving monocyte/macrophages and improved recovery in elderly patients [139].

## Conclusions

It is possible that in the elderly due to the inflammageing microenvironment, compensatory regulatory mechanisms exist which subsequently delay repair following injury and promote tumor growth. This could further impact drugs designed to target inflammatory and regulatory immune cell subsets, including macrophages, for clinical use in elderly humans. This is of particular importance given the number of studies which suggest that overcompensation of immune responses potentially leads to immune dysregulation during ageing. As highlighted above, further studies are required to understand the cross-talk between macrophages and other immune cells during ageing, along with the impact of tissue-specific changes and sex on macrophage responses following musculoskeletal injury and cancer. Whilst recent studies have described changes to macrophages during healthy ageing, it is clear that further studies are required to elucidate the underlying mechanisms behind changes to macrophage function, in both lymphoid and other tissues, during age-related diseases and co-morbidities.

## Data Availability

Not applicable.

## Abbreviations

CCL: C-C motif ligand
CCR2: C-C chemokine receptor 2
CSF-1: Colony stimulating factor-1
CSF-1R: Colony stimulating factor-1 receptor
CXCL: C-X-C motif ligand
CXCR2: C-X-C chemokine receptor 2
IFN-γ: Interferon-gamma
IL: Interleukin
JAK: Janus kinase
LPS: Lipopolysaccharide
MAPK: Mitogen-activated protein kinases
MDSCs: Myeloid-derived suppressor cells
NAD: nicotinamide adenine dinucleotide
STAT: Signal transducers and activators of transcription
TGF-β1: Transforming growth factor-beta1
TNF: Tumor necrosis factor
Tregs: Regulatory CD4^+^ T cells
